# Nasal and Salivary Mucosal Humoral Immune Response Elicited by mRNA BNT162b2 COVID-19 Vaccine Compared to SARS-CoV-2 Natural Infection

**DOI:** 10.3390/vaccines9121499

**Published:** 2021-12-18

**Authors:** Mariapia Guerrieri, Beatrice Francavilla, Denise Fiorelli, Marzia Nuccetelli, Francesco Maria Passali, Luca Coppeta, Giuseppina Somma, Sergio Bernardini, Andrea Magrini, Stefano Di Girolamo

**Affiliations:** 1 Department of Otorhinolaryngology, University of Rome “Tor Vergata”, 00100 Rome, Italy; Mariapia.guerrieri@ptvonline.it (M.G.); passali@med.uniroma2.it (F.M.P.); stefano.di.girolamo@uniroma2.it (S.D.G.); 2 Department of Experimental Medicine, University of Rome “Tor Vergata”, 00100 Rome, Italy; denise.fiorelli@ptvonline.it (D.F.); Marzia.nuccetelli@ptvonline.it (M.N.); sergio.bernardini@ptvonline.it (S.B.); 3 Department of Occupational Medicine, University of Rome “Tor Vergata”, 00100 Rome, Italy; luca.coppeta@ptvonline.it (L.C.); giuseppina.somma@ptvonline.it (G.S.); andrea.magrini@ptvonline.it (A.M.); 4 Department of Laboratory Medicine, Tor Vergata University Hospital, 00100 Rome, Italy

**Keywords:** IgA, nasal, salivary, SARS-CoV-2, vaccine, mucosal, immunity, BNT162b2, COVID-19, IgG-RBD

## Abstract

SARS-CoV-2 antibody assays are crucial in managing the COVID-19 pandemic. Approved mRNA COVID-19 vaccines are well known to induce a serum antibody responses against the spike protein and its RBD. Mucosal immunity plays a major role in the fight against COVID-19 directly at the site of virus entry; however, vaccine abilities to elicit mucosal immune responses have not been reported. We detected anti-SARS-CoV-2 IgA-S1 and IgG-RBD in three study populations (healthy controls, vaccinated subjects, and subjects recovered from COVID-19 infection) on serum, saliva, and nasal secretions using two commercial immunoassays (ELISA for IgA-S1 and chemiluminescent assay for IgG-RBD). Our results show that the mRNA BNT162b2 vaccine Comirnaty (Pfizer/BioNTech, New York, NY, USA) determines the production of nasal and salivary IgA-S1 and IgG-RBD against SARS-CoV-2. This mucosal humoral immune response is stronger after the injection of the second vaccine dose compared to subjects recovered from COVID-19. Since there is a lack of validated assays on saliva and nasal secretions, this study shows that our pre-analytical and analytical procedures are consistent with the data. Our findings indicate that the mRNA COVID-19 vaccine elicits antigen-specific nasal and salivary immune responses, and that mucosal antibody assays could be used as candidates for non-invasive monitoring of vaccine-induced protection against viral infection.

## 1. Introduction

The mucosal humoral immune response has a pivotal role in the fight against novel Coronavirus Disease (COVID-19) [1,2,3]. As SARS-CoV-2 primarily infects the upper respiratory tract, the first interactions with the immune system of the host take place on nasal and oropharyngeal mucosa, where specific secretory immunoglobulins are capable of counteracting the infection [4]. The mucosal immune system is the first to respond to the virus, producing secretory antibodies that can be detected in upper respiratory tract secretions [5]. In particular, secretory IgA (S-IgA), located on the mucous membranes, plays a crucial role in mucosal immunity. In fact, this antibody can neutralize pathogens, particularly in respiratory tract infections caused by viruses, protecting the local mucosa from viral invasion [6].

SARS-CoV-2 infection results in a wide range of clinical signs, varying from asymptomatic to life-threatening acute respiratory distress syndrome, which is caused by a deleterious antiviral immune response in lungs [7]. Some subjects develop mild symptoms of COVID-19, localized in the upper respiratory tract (rhinitis with rhinorrhea, anosmia, and ageusia) without severe pulmonary involvement [8]. This suggests the important role played by mucosal immunity: secretory antibodies and in particular S-IgA can neutralize SARS-CoV-2 before it reaches and binds to the epithelial cells, acting as an immune barrier [1].

Previous studies have shown that antibodies in the respiratory tract or oral cavity could be important for protecting against other human respiratory viruses like SARS-CoV, influenza virus, and respiratory syncytial virus (RSV) [6,9]. Nevertheless, in the fight against COVID-19, most attention has been given to circulating virus-neutralizing antibodies, especially IgG and IgM [10,11,12,13,14]. Previous studies have demonstrated that in the first weeks after symptom onset, SARS-CoV-2 systemic neutralization is correlated more closely with IgA than with IgM or IgG. However, these can all be effective in the prevention of infection or disease if they reach the mucosal surfaces where the virus is present [1,4]. Secretory IgA (S-IgA) is the principal antibody class present in mucosal surfaces, produced as dimeric IgA by local plasma cells. On these surfaces it is possible to also find mucosal IgG, mostly derived from blood circulation by passive leakage. This originates in part via gingival crevicular epithelium, although some may be locally produced [15]. Preliminary studies have shown that adult subjects with acute COVID-19 have high levels of specific and neutralizing S-IgA detectable in the saliva [3,16,17,18,19,20].

The SARS-CoV-2 spike (S) protein plays the most critical role in viral attachment, fusion, and entry into the target cell [21,22,23]. The S protein is divided into two functional subunits, S1 and S2. Subunit S1 is responsible for binding to the host cell receptor (angiotensin-converting enzyme 2 receptor ACE-2) through its receptor-binding domain (RBD) [5]. The S2 subunit contains the necessary elements required for membrane fusion [24,25]. During infection, SARS-CoV-2 first binds the host cell through interaction between its S1-RBD and the cell membrane receptor, triggering conformational changes in the S2 subunit that result in virus fusion and entry into the target cell [24,25]. The ACE-2 receptor for SARS-CoV-2 cellular entry is most highly expressed in the upper respiratory tract, and most SARS-CoV-2 shedding occurs from the upper respiratory tract [14]. Among the SARS-CoV-2 proteins, RBD seems to be the most antigenic protein with a neutralizing activity [26].

SARS-CoV-2 antibody assays are relevant in managing the COVID-19 pandemic, as they provide valuable data on the immunization status of the population [12,27,28]. Reported validated serology tests to assess immunogenicity focus on anti-SARS-CoV-2 circulating anti-spike IgG antibodies, which include IgG, against the receptor binding domain (RBD), the subunit 1 (S1), or the full spike (S) [29]. Nevertheless, several different serological immunoassays have been proposed on the market, resulting in a difficult comparison of data. Recently, an international standard unit of measurement, named the Binding Antibody Unit (BAU), was introduced by the WHO to harmonize results [28]. 

The approved SARS-CoV-2 mRNA vaccines, BNT162b2 Comirnaty (Pfizer/BioNTech, New York, NY, USA) and Spikevax (Moderna, Cambridge, MA, USA) are proven to induce a serum IgG humoral response predominantly directed against the SARS-CoV-2 RBD region of the S1 Spike protein subunit [24,30]. However, the humoral response to COVID-19 vaccination remains largely unpredictable [30], and vaccine abilities to elicit mucosal immune responses have not yet been reported. Regarding vaccine application, several studies have highlighted the utility of antibody assays as a highly cost-effective tool for a vaccination strategy to promote public health [31,32,33]. At this purpose, standardized anti-SARS-CoV-2 quantitative and neutralizing assays are pivotal in assessing immune responses to vaccines, and they will become essential if a correlation between the antibody concentration and the protection threshold could be identified [28]. 

Recently, it has been demonstrated that the intranasal administration by adenovirus-vectored vaccine encoding spike protein induced robust IgA and neutralizing antibody responses and prevented SARS-CoV-2 infection to both the upper and lower respiratory tract [6,34]. To this end, nasal and salivary antibodies could be relevant to demonstrate mRNA vaccines effects on the prevention of oral and nasal SARS-CoV-2 acquisition and transmission [16]. The amount of specific anti-spike IgA and IgG in the respiratory and oropharyngeal mucosa may thus serve as an indicator of host immune response and protection [5]. Nevertheless, at present, there are a lack of validated tests to assess the mucosal response to COVID-19 vaccination and to define standardized analytic and pre-analytic methods.

For this purpose, to compare the natural and vaccine-acquired systemic and mucosal humoral responses, in our study, we detected anti-SARS-CoV-2 IgA-S1 and IgG-RBD in three study populations (healthy controls, vaccinated subjects, and COVID-19 subjects) on serum, saliva, and nasal secretions samples with two commercial immunoassays (enzyme-linked immunosorbent assay for IgA-S1 and chemiluminescent assay for IgG-RBD). 

## 2. Materials and Methods

### 2.1. Study Population and Setting

The study was conducted at the Tor Vergata University Hospital in Rome, Italy, and included samples collected between April and June 2021. 

Subjects were divided into three groups (Table 1). The first group (healthy controls, T0) included 33 subjects that never contracted natural SARS-CoV-2 infection, and who were enrolled immediately before the first injection of the mRNA BNT162b2 COVID-19 vaccine Comirnaty (Pfizer/BioNTech, New York, NY, USA). 

The second group (vaccinated subjects, T1) included 28 subjects from the first group who agreed to be tested again 15 days after the injection of the second dose of the mRNA BNT162b2 COVID-19 vaccine (Comirnaty). The third group (COVID-19 population) included 18 subjects who recovered from previous SARS-CoV-2 infection, with a positive quantitative reverse-transcriptase polymerase chain reaction (RT-qPCR) within the previous 70 days (mean time from the first RT-qPCR+: 49.5 days; mean time from the first RT-qPCR−: 31.4 days). At the time of the study, none of the COVID-19 subjects had residual symptoms (except for hyposmia). Fifteen subjects in this group reported a mild or pauci-symptomatic infection, while three subjects had severe pneumonia that required hospitalization. 

Peripheral blood, nasal secretions, and saliva samples were collected for each subject. In total, 79 samples of saliva, nasal secretions, and serum were collected: 51 adult subjects (26 F and 25 M, median age 49 y, range 22–70 years) were enrolled in the first and in the third group, and 28 of these subjects (14 F and 14 M, median age 52 years) were retested again in the second group. At the time of enrolment, none of the subjects had an acute infection, serious illness, or were taking any medication known to alter immune function.

The study was submitted to the Ethical Committee of the Tor Vergata University Hospital (protocol number 9962/2021).

### 2.2. Collection of Samples

Following written informed consent, subjects were recruited for the sampling of blood, nasal fluids, and saliva. 

Nasal secretions were collected by the insertion of cotton wool into both nasal cavities for ten minutes, then hydrating for ten minutes with 10 mL of 0.9% NaCl saline solution, and subsequently squeezing through a syringe into a test tube and storing in Eppendorf tubes at −80 °C. 

Saliva was collected using a cotton roll (Neutral Salivette^®^, SARSTEDT, Numbrecht, Germany) held for 1 min in the mouth of the participant, prior to replacing it into the stopper part of the Salivette^®^ tube. The samples were centrifuged at 1500× *g* for 5 min at 4 °C, and the resulting saliva was stored in Eppendorf tubes at −80 °C. 

The serum was collected by peripheral venous sampling in dry tubes, then centrifuged for 15 min at 4000× *g*, and was stored in special Eppendorf tubes at a controlled temperature of −80 °C.

### 2.3. Biochemical Analysis

#### 2.3.1. CLIA for IgG-RBD Detection

MAGLUMI^®^ SARS-CoV-2 S-RBD IgG by SNIBE Diagnostic (Shenzhen, China) is an indirect chemiluminescent immunoassay designed for the quantitative detection of IgG anti-RBD levels in the serum or plasma samples, using the fully automated MAGLUMI 800 analyzer. It can be used to characterize the immune response of COVID-19 subjects and individuals who have been vaccinated against the virus, representing an important tool for assessment of the efficacy of COVID-19 vaccines. 

Serum samples were used in combination with sample buffer and magnetic particles coated with RBD antigen and were subsequently incubated in order to promote immune complexes. The supernatant was removed after sedimentation in a magnetic field. Anti-Human IgG antibodies labeled with ABEI (amino-butyl-ethyl-isoluminol) were added after washing and after incubation and, additional washing, the Starter 1 + 2 solution was added. The emitted light is measured as relative light units (RLUs), and RLUs are directly proportional to the corresponding IgG anti-RBD concentration in the samples. 

The results were reported in AU/mL and the manufacturer’s recommended cut-off value in the serum is >1.00 AU/mL. To standardize data and to convert the results into Binding Antibody Units (BAU/mL), the SNIBE diagnostic provided the following unit conversion relationships: 1 AU/mL is equivalent to 4.33 BAU/mL (BAU = AU × 4.33). Therefore, the cut-off value was >4.33 BAU/mL. 

The cut-offs obtained in our healthy control study populations were 1.19 BAU/mL and 0.63 BAU/mL in the saliva and nasal fluids, respectively.

All data in the present study are reported in BAU/mL.

#### 2.3.2. ELISA for S1-IgA Detection

Immunoglobulins Class A (IgA) against SARS-Cov-2 spike S1 in the serum, nasal secretions, and saliva were identified by an ELISA test using a well plate coated with recombinant S protein antigen (Euroimmun Medizinische Laboradiagnostika, Luebeck, Germany). Sample testing was performed automatically using the Euroimmun analyzer instrumentation (Euroimmun Medizinische Laboradiagnostika, Luebeck, Germany). 

According to the manufacturer’s instructions, the serum samples were diluted 1:101 in the sample buffer; the best performance dilutions for the nasal secretion samples and saliva were 1:2 dilution and 1:50 dilution, respectively. Positive and negative controls, diluted subject samples, and a calibrator to obtain the cut-off values were transferred to the wells of the microplate and were incubated for 60 min at +37 °C ± 1 °C. 

After washing, 100 μL of enzyme conjugate (anti-human IgA labeled with peroxidase) was added and incubated for 30 min at +37 °C ± 1 °C. Finally, 100 μL of the chromogen substrate (TMB (tetra-methyl-benzidine)) solution was added, developing a colorimetric reaction. The optical density was proportional to the quantity of the specific anti-SARS-CoV-2 IgA antibodies present in the samples. 

The results were calculated semi-quantitatively by a ratio between the sample OD and the calibrator OD. The serum cut-offs were considered negative for all the values < 0.8 COI (cut-off index), equivocal for all the values between 0.8 and 1.1 COI and positive for all the values >1.1 COI, as declared by the manufacturer. 

The cut-offs obtained in our healthy controls study populations are 10.50 and 0.86 COI in the saliva and nasal fluids, respectively. 

### 2.4. Data Analysis and Statistics

Statistical analysis and construction of figures were performed with GraphPad Prism 8 Software (GraphPad Software, San Diego, CA, USA). The D’Agostino and Pearson test, Shapiro–Wilk normality test, and Kolmogorov–Smirnov test were used to evaluate the non-Gaussian distributions in all of the study populations. The categorical data were displayed as numbers and/or percentages and continuous data as median and range. Non-parametric results were analyzed with the Mann–Whitney test. 

For all of the results, *p* < 0.05 was considered statistically significant.

## 3. Results

**Figure 1:**
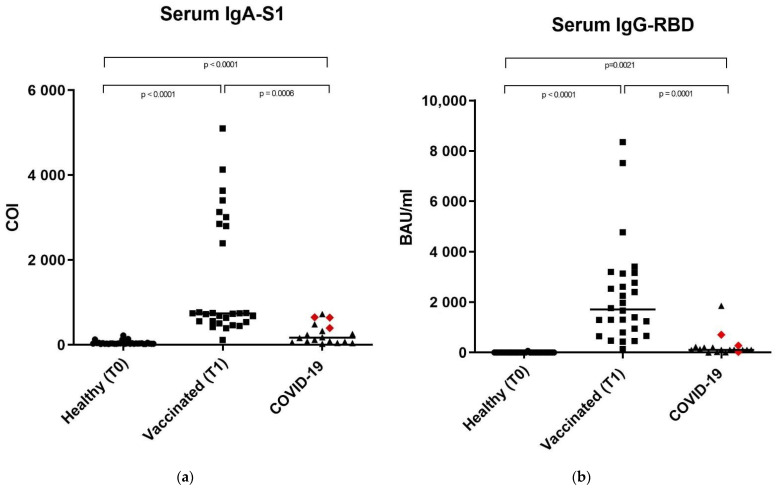
Anti SARS-CoV-2 IgA-S1 and anti SARS-CoV-2 IgG-RBD in serum samples. (**a**) Serum sample median levels of anti SARS-CoV-2 IgA-S1 in the three study groups expressed as COI (Cut off index). (**b**) Serum sample median levels of anti SARS-CoV-2 IgG-RBD in the three study groups, expressed as Binding Antibody Units (BAU/mL). In the COVID-19 group, the red rhombuses represent the hospitalized subjects. Statistical analysis and construction of figures were performed with GraphPad Prism 8 Software (GraphPad Software, San Diego, CA, USA). The D’Agostino and Pearson test, Shapiro–Wilk normality test, and Kolmogorov–Smirnov test were used to evaluate non-Gaussian distributions in all study populations. The continuous data were displayed as median and range. Non-parametric results were analyzed with the Mann–Whitney test. For all of the results, *p* < 0.05 was considered statistically significant.

**Figure 2:**
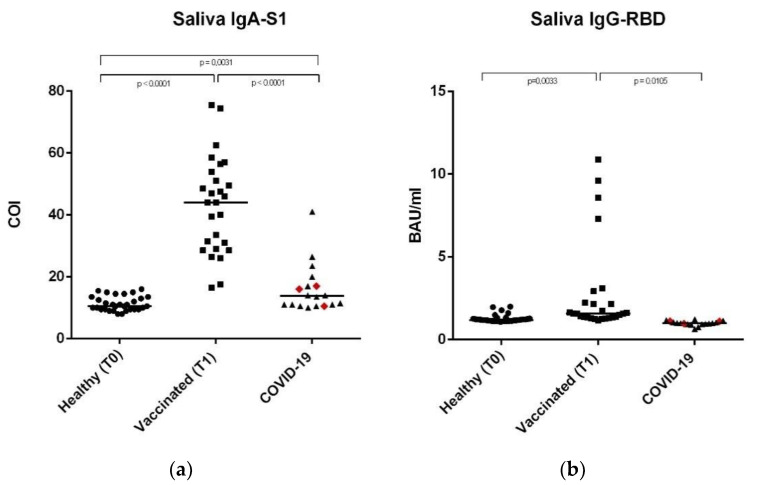
Anti-SARS-CoV-2 IgA-S1 and anti-SARS-CoV-2 IgG-RBD in saliva samples. (**a**) Saliva sample median levels of anti-SARS-CoV-2 IgA-S1 in the three study groups, expressed as COI (Cut off index). (**b**) Saliva sample median levels of anti-SARS-CoV-2 IgG-RBD in the three study groups, expressed as Binding Antibody Units (BAU/mL). In the COVID-19 group, the red rhombuses represent the hospitalized subjects. Statistical analysis and construction of figures were performed with GraphPad Prism 8 Software (GraphPad Software, San Diego, CA, USA). The D’Agostino and Pearson test, the Shapiro-Wilk normality test, and the Kolmogorov–Smirnov test were used to evaluate non-Gaussian distributions in all of the study populations. The continuous data were displayed as median and range. Non-parametric results were analysed with the Mann–Whitney test. For all results, *p* < 0.05 was considered statistically significant.

**Figure 3:**
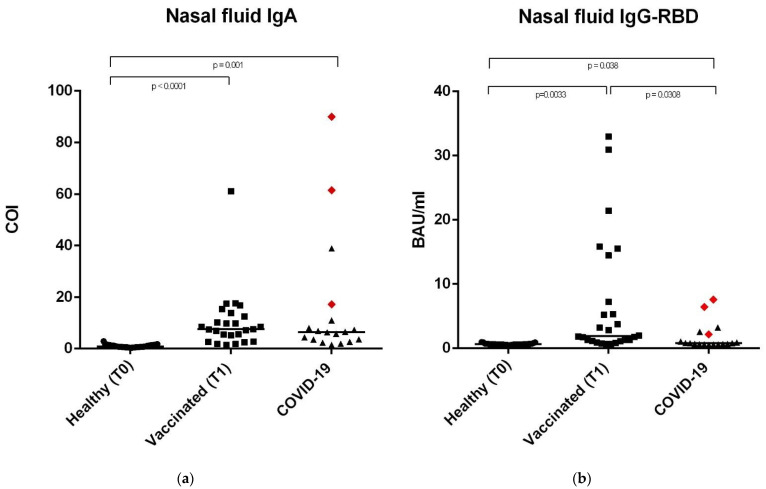
Anti SARS-CoV-2 IgA-S1 and anti SARS-CoV-2 IgG-RBD in nasal fluid samples. (**a**) Nasal fluid sample median levels of anti SARS-CoV-2 IgA-S1 in the three study groups, expressed as COI (Cut off index). (**b**) Nasal fluid sample median levels of anti SARS-CoV-2 IgG-RBD in the three study groups, expressed as Binding Antibody Units (BAU/mL). In the COVID-19 group, the red rhombuses represent the hospitalized subjects. Statistical analysis and construction of figures were performed with GraphPad Prism 8 Software (GraphPad Software, San Diego, CA, USA). The D’Agostino and Pearson test, Shapiro–Wilk normality test, and Kolmogorov– Smirnov test were used to evaluate the non-Gaussian distributions in all of the study populations. The continuous data were displayed as median and range. Non-parametric results were analysed with the Mann–Whitney test. For all of the results, *p* < 0.05 was considered statistically significant.

Anti-SARS-CoV-2 IgA-S1 and IgG-RBD antibodies have been detected on the serum and saliva samples, as well as on nasal secretions of 33 healthy controls, 28 vaccinated subjects, and 18 subjects recovered from COVID-19. This last group included 15 subjects with previous mild or pauci-symptomatic infection and three subjects recovered from a severe infection that required hospitalization, represented in Figure 1, Figure 2 and Figure 3 as red rhombuses.

As validated assays for anti-SARS-CoV-2 antibody detection on saliva and nasal solutions are not currently available, our procedural approach provided results compatible with the three different groups. 

### 3.1. Anti-SARS-CoV-2 IgA-S1 and IgG-RBD in Serum Samples

The median serum concentrations reported in Figure 1 and Table 2 showed the same trend for anti-SARS-CoV-2 IgA-S1 and IgG-RBD, with higher values in the vaccinated group (739.3 COI and 1711 BAU/mL, IgA-S1 and IgG-RBD, respectively) and progressively lower values in the COVID-19 subjects (169.70 COI and 109.10 BAU/mL for IgA and IgG-RBD, respectively) and healthy controls (29.29 COI and 0.75 BAU/mL for IgA and IgG-RBD, respectively). Data were statistically significant with a *p*-value <0.0001 between IgA-S1 in the healthy vs. COVID-19 groups, IgA-S1 in the healthy vs. vaccinated populations, and IgG-RBD in the healthy vs. vaccinated groups. Data were statistically significant with a *p*-value = 0.001 between IgA-S1 and IgG-RBD in the vaccinated vs. COVID-19 groups, except for the comparison between IgG-RBD in the healthy vs. COVID-19 populations, which had a *p*-value = 0.0021.

### 3.2. Anti-SARS-CoV-2 IgA-S1 and IgG-RBD in Saliva Samples

In the saliva samples, the median anti-SARS-CoV-2 IgA concentrations were higher in the vaccinated group (44 COI) compared to the COVID-19 subjects and healthy controls, where the values were similar (13.75 vs. 10.50, respectively; considering 10.50 COI as the cut-off value). Data were statistically significant: *p*-value < 0.001 between COVID-19 subjects vs. vaccinated subjects and between the vaccinated group vs. healthy controls (Figure 2a and Table 3); *p* value = 0.0031 between the healthy controls vs. COVID-19 subjects.

Different results were obtained for anti-SARS-CoV-2 IgG-RBD concentrations where, as expected, data were extremely low (1.57, 1.19, and 1.00 BAU/mL in the vaccinated, COVID-19, and control groups, respectively), considering 1.19 as the cut-off value. These results showed a different trend when compared to the results obtained in all of the other samples. Data were statistically significant, with a *p*-value = 0.0033 between healthy and vaccinated subjects, *p* = 0.011 between COVID-19 subjects vs. the vaccinated group, and *p* = 0.033 between the vaccinated group vs. controls (Figure 2b and Table 3).

### 3.3. Anti-SARS-CoV-2 IgA-S1 and IgG-RBD in Nasal Secretions

Finally, the anti-SARS-CoV-2 IgA-S1 and IgG-RBD in the nasal secretions followed a similar trend. The anti-SARS-CoV-2 IgG-RBD in the nasal solutions showed higher values in the vaccinated subjects (1.19 BAU/mL) compared to the COVID-19 subjects (0.79 BAU/mL) and to healthy controls (0.63 BAU/mL), considering 0.63 BAU/mL as the cut-off value. Data were statistically significant with a *p*-value = 0.0038 between vaccinated subjects (*p* = 0.0033), a *p*-value of 0.038 in COVID-19 subjects vs. healthy controls, and *p* = 0.031 between vaccinated group vs. controls) (Table 4 and Figure 3b). 

IgA-S1 in nasal secretions showed higher median IgA values in vaccinated and COVID-19 subjects (7.56 and 6.44 COI, respectively) compared to the healthy controls (0.86 COI) (Table 4 and Figure 3a) (*p*-value = 0.001 between COVID-19 subjects vs. healthy controls and *p* < 0.0001 between vaccinated subjects vs. healthy controls), considering 0.86 COI as the cut-off value. 

### 3.4. Anti-SARS-CoV-2 IgA-S1 and IgG-RBD Values in Subjects with Mild vs. Severe COVID-19 Infection

**Figure 4:**
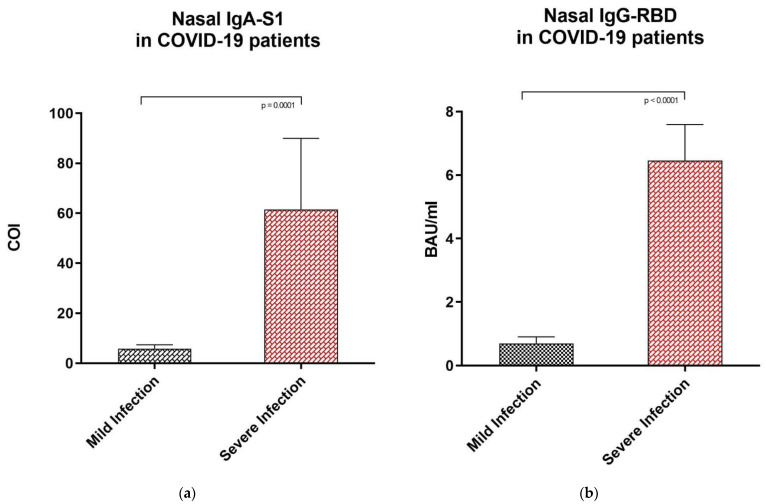
Anti SARS-CoV-2 IgA-S1 and anti SARS-CoV-2 IgG-RBD in nasal fluid samples of subjects recovered from previous mild and severe COVID-19 infection. (**a**) Nasal secretions median levels of anti SARS-CoV-2 IgA-S1 in subjects with previous mild COVID-19 infection and severe infection, expressed as COI (Cut off index). (**b**) Nasal fluid samples median levels of anti SARS-CoV-2 IgG-RBD in subjects with previous mild COVID-19 infection and hospitalized subjects, respectively, expressed as Binding Antibody Units (BAU/mL). Statistical analysis and construction of figures were performed with GraphPad Prism 8 Software (GraphPad Software, San Diego, CA, USA). The D’Agostino and Pearson test, Shapiro–Wilk normality test, and Kolmogorov–Smirnov test were used to evaluate non-Gaussian distributions in all study populations. The error bars report the median, and 75° and 25° percentiles. The non-parametric results were analysed with the Mann–Whitney test. For all of the results, *p* < 0.05 was considered statistically significant.

Our results showed significant differences in antibody levels among the COVID-19 group between subjects with severe (*n* = 3, represented as red rhombuses in Figure 1, Figure 2 and Figure 3) vs. mild or pauci-symptomatic (*n* = 15) infection. Subjects with previous severe SARS-CoV-2 infection that required hospitalization showed higher anti-SARS-CoV-2 IgA and IgG-RBD values in the serum samples (643.4 COI vs. 118.2 COI and 275.0 BAU/mL vs. 108.6 BAU/mL, respectively) and in nasal secretions (61.5 COI vs. 5.78 COI and 6.46 BAU/mL vs. 0.7 BAU/mL, respectively), as shown in Figure 4. 

Interestingly, in nasal secretions, the median values of the anti-SARS-CoV-2 IgA-S1 and IgG-RBD in subjects with previous severe COVID-19 infection were also higher than the median values of vaccinated subjects (61.50 COI vs. 7.56 COI and 6.46 BAU/mL vs. 1.91 BAU/mL, respectively), as shown in Table 4 and Table 5. On the other hand, the previous severe COVID-19 subject serum sample median values of anti-SARS-CoV-2 IgA-S1 and IgG-RBD were lower than the median antibody concentrations in vaccinated subjects (IgA-S1 643.4 COI vs. 739.30 COI; IgG-RBD 275.0 BAU/mL vs. 1711.00 BAU/mL). 

## 4. Discussion

Coronavirus disease 2019 (COVID-19) is characterized by heterogeneity in susceptibility to the disease, immune response, and severity of illness. Understanding interindividual variation has important implications, not only for the allocation of resources, but also for targeting subjects for the escalation of care and individualized medical therapy, including vaccination [35].

The correlates of vaccine-induced immunity are a subject of continuous interest for both theoretical and practical reasons. After the administration of almost all of the vaccines, the prevention of infection correlates with the induction of specific antibodies. However, as reported by Plotkin [36], antibodies must be present at the site of replication on the mucosae to provide protection from infection. 

SARS-CoV-2 salivary and nasal immune response remain poorly documented and little is known about vaccine-elicited mucosal humoral responses. Few studies have shown that specific IgA are detectable in saliva samples during acute SARS-CoV-2 infection [2,16,17], persisting for at least three months after infection [37]. Ketas et al. [38] described preliminary evidence that anti-SARS-CoV-2 IgA are also detectable in the saliva samples from mRNA-vaccinated healthcare workers. Moreover, Piano Mortari et al. reported that memory B cells migrate in response to inflammation and secrete IgA at mucosal sites, even without evidence of the presence of mucosal IgA induced by COVID-19 vaccination [39]. 

Froberg et al. [40] recently reported a significant increase in nasal antibody levels in response to COVID-19 acute infection, with similar kinetics as described for the serum and saliva. Nevertheless, as the nasal immune response induced by the SARS-CoV-2 vaccine has not yet been studied, the standard method for antibody quantification in nasal secretions and saliva samples still needs to be further clarified. 

Our results show that the mRNA BNT162b2 COVID-19 vaccine Comirnaty (Pfizer/BioNTech, New York, NY, USA) determines the production of both circulating and mucosal specific IgA and IgG, directed against the S1-protein and the receptor binding domain (RBD) of SARS-CoV-2, respectively. Finding secretory antibodies against SARS-CoV-2 in the saliva and nasal secretions of people that only received the vaccine, without natural infection, is an unexpected yet intriguing evidence, as it allows for surmising that the vaccine can offer a barrier against infection directly at the site of virus entry.

Comparison of natural and vaccine-acquired humoral responses could help in understanding the effective potential of COVID-19 vaccines. As there is a lack of validated assays on saliva and nasal secretions, this study showed that our pre-analytical and analytical procedures are consistent with the data. In fact, in healthy controls anti-SARS-CoV-2 IgA-S1 and IgG RBD were not measurable or were significatively lower in comparison with vaccinated subjects and COVID-19 subjects. 

Our results showed, in all the biological fluids analyzed, higher anti-SARS-CoV-2 IgA-S1 and IgG-RBD production in vaccinated subjects in comparison with healthy controls and subjects recovered from previous COVID-19. Interestingly, as vaccinated individuals coincided with the healthy control group, these findings indicate that vaccination increased not only the IgG-RBD levels, but also the IgA-S1 levels in all of the analyzed matrices. 

Subjects that had recovered from a previous mild or asymptomatic SARS-CoV-2 infection showed lower levels of mucosal and serological antibodies in comparison with vaccinated subjects. This was particularly visible in the serum samples, consistent with previous studies. Notably, this difference was evident also when comparing IgA-S1 in the saliva and nasal secretions of vaccinated and mild COVID-19 subjects. The three subjects that had recovered from a severe COVID-19 infection that required hospitalization showed higher levels of circulating and mucosal antibodies in all of the analyzed samples in comparison with subjects with previous mild or pauci-symptomatic infection. This difference between the anti-SARS-CoV-2 antibody levels in subjects with mild and severe infection may probably be ascribed to the different viral load in the two conditions [40,41]. Interestingly, the levels of circulating and mucosal antibodies in subjects with previous mild or pauci-symptomatic infection were comparable to the amount present in the vaccinated population. In particular, these subjects showed increased levels of IgA-S1 in nasal secretions, similarly to the vaccinated subjects.

The current paradigm in vaccine development is that nonreplicating vaccines delivered parenterally fail to induce immune responses in the mucosal tissues. However, both clinical and experimental preliminary data have challenged this concept [42]. Finding secretory anti-SARS-CoV-2 IgA-S1 in the saliva and nasal secretions after systemic vaccination with a nonreplicating vaccine was an unexpected and paradigm-shifting result. Our results showed that the induction of mucosal immune responses after systemic vaccination might significantly contribute to protection against mucosal infection. 

The presence of salivary anti-SARS-CoV-2 IgA following intramuscular immunization with the mRNA BNT162b2 COVID-19 vaccine confirmed what was preliminarily reported by Ketas et al. [38], whereas having added the evaluation of nasal secretions is the novelty of this study. To the best of our knowledge, this is the first paper reporting a vaccine-elicited SARS-CoV-2 nasal immune response. Moreover, interestingly, we did not find only anti-SARS-CoV-2 IgA-S1, the main antibody class in mucosal surfaces, but also anti-SARS-CoV-2 IgG-RBD in the nasal secretions of vaccinated subjects. This could be due to exudation from the blood [15] or it could be explained considering a specific function of the spike protein on capillary permeabilization. Further studies are required to explain the mechanisms underlying this finding. 

Recently, Lippi et al. [31] highlighted the potential advantages of serological testing in recipients of COVID-19 vaccinations: assessment of the baseline seroprevalence of SARS-CoV-2 infection in non-vaccinated individuals, the identification of low- or non-responders to the COVID-19 vaccine, and the timely detection of the faster decay of anti-SARS-CoV-2 antibody. In contrast, potential drawbacks to date include the enormous volume of blood drawings and the increase of laboratory workload needed to support anti-SARS-CoV-2 serological antibody testing [34].

Finding out if current vaccines could induce a specific mucosal humoral immune response against SARS-CoV-2, alongside the systemic one, would provide an important hint towards the end of this global pandemic. Our findings indicate that the COVID-19 vaccine can induce a humoral immune response in the mucosa and mucosal, antibody assays could be used as candidates for non-invasive and cost-effective monitoring of vaccine-induced protection against viral infection. 

## 5. Conclusions

In conclusion, our data demonstrate that the mRNA BNT162b2 COVID-19 vaccine (Comirnaty) elicits an antigen-specific mucosal immune response. 

Significant levels of secretory IgA against the S1 protein can be found in nasal secretions and saliva after the injection of the second vaccination dose. This mucosal humoral immune response is more relevant in vaccinated subjects in comparison with subjects recovered from a pauci-symptomatic or mild SARS-CoV-2 natural infection. Only subjects that had recovered from severe COVID-19 infection requiring hospitalization had higher levels of S-IgA at the mucosal sites, which were comparable with the vaccinated subjects. As there are no validated assays for saliva and in particular for nasal secretions, with our study, we propose an analytical and pre-analytical procedure that can give consistent data to evaluate the specific mucosal IgA and IgG, respectively, directed against the SARS-CoV-2 S1-protein and its RBD. 

Our findings suggest that monitoring of the humoral mucosal immunity directly at the site of virus entry could be an effective way to assess a vaccine-induced immune response and to evaluate the timing of the antibody decay and the effective individual protection. Further studies are required to determine if mucosal assays could address public health decisions, such as the individual timing of subsequent dose injections and the persistent requirement of face protections. 

## Figures and Tables

**Table 1:** Populations studied.

Population	Vaccine	SARS-CoV-2 Infection
COVID-19 (*n* = 18)	Non-vaccinated	Recovered from previous COVID-19 (RT-qPCR+ <70 days), no longer reporting symptoms
Healthy controls T0 (*n* = 33)	Non-vaccinated, enrolled immediately before the 1st *mRNA BNT126b2b* vaccine injection	Subjects with no previous SARS-CoV-2 infection
Vaccinated T1 (*n* = 28)	Vaccinated: subjects of the healthy control group, retested 15 days after the 2nd *mRNA BNT126b2b* vaccine injection	Subjects with no previous SARS-CoV-2 infection

**Table 2:** Serum sample range, median, and dilution values of anti SARS-CoV-2 IgG-RBD and anti SARS-CoV-2 IgA-S1 antibody levels in the three study groups.

SERUM				
	Population	Median	Range	Dilution
IgG-RBD	Healthy	0.75 BAU/ml	0.46–66.77 BAU/ml	No dilution
	Vaccinated	1711.00 BAU/ml	144.80–8352.00 BAU/ml	1:100
	COVID-19	109.10 BAU/ml	3.25–1853.00 BAU/ml	1:100
IgA-S1	Healthy	29.29 COI	20.20–219.20 COI	1:101
	Vaccinated	739.30 COI	116.20–5101.0 COI	1:101
	COVID-19	169.70 COI	26.26–725.20 COI	1:101

**Table 3:** Saliva samples range, median, and dilution values of anti SARS-CoV-2 IgG-RBD and anti SARS-CoV-2 IgA-S1 antibody levels in the three study groups.

SALIVA				
	Population	Median	Range	Dilution
IgG-RBD	Healthy	1.19 BAU/ml	1.08–2.00 BAU/ml	No dilution
	Vaccinated	1.57 BAU/ml	1.15–10.88 BAU/ml	No dilution
	COVID-19	1.00 BAU/ml	0.65–1.23 BAU/ml	No dilution
IgA-S1	Healthy	10.50 COI	8.00–16.00 COI	1:50
	Vaccinated	44.00 COI	16.50–75.50 COI	1:50
	COVID-19	13.75 COI	10.00–41.00 COI	1:50

**Table 4:** Nasal secretions range, median, and dilution values of anti-SARS-CoV-2 IgA-S1 and anti-SARS-CoV-2 IgG-RBD antibody levels in the three study groups.

NASAL SECRETIONS				
	Population	Median	Range	Dilution
IgG-RBD	Healthy	0.63 BAU/ml	0.48–0.99 BAU/ml	No dilution
	Vaccinated	1.91 BAU/ml	0.65–32.97 BAU/ml	No dilution
	COVID-19	0.79 BAU/ml	0.57–7.59 BAU/ml	No dilution
IgA-S1	Healthy	0.86 COI	0.32–2.92 COI	1:2
	Vaccinated	7.56 COI	1.40–61.10 COI	1:2
	COVID-19	6.44 COI	1.44–90.00 COI	1:2

**Table 5:** Range and median values of anti SARS-CoV-2 IgA-S1 and anti SARS-CoV-2 IgG-RBD antibody levels in nasal secretions in subjects with previous COVID-19 mild infection vs. subjects with previous severe infection that required hospitalization.

NASAL SECRETIONS OF COVID-19 SUBJECTS				
	Population	Median	Range	Dilution
IgG-RBD	Mild or pauci-symptomatic infection	0.70 BAU/ml	0.57–3.25 BAU/ml	No dilution
	Severe infection	6.46 BAU/ml	2.17–7.59 BAU/ml	No dilution
IgA-S1	Mild or pauci-symptomatic infection	5.78 COI	1.44–38.90 COI	1:2
	Severe infection	61.50 COI	17.24–90.00 COI	1:2

## Data Availability

The data that support the findings of this study are available from the corresponding author upon reasonable request.

## References

[1] Chao Y.X., Rötzschke O., Tan E.K. (2020). The role of IgA in COVID-19. Brain Behav. Immun..

[2] Cervia C., Nilsson J., Zurbuchen Y., Valaperti A., Schreiner J., Wolfensberger A., Raeber M.E., Adamo S., Weigang S., Emmenegger M. (2021). Systemic and mucosal antibody responses specific to SARS-CoV-2 during mild versus severe COVID-19. J. Allergy Clin. Immunol..

[3] Pearson C.F., Jeffery R., Thornton E.E., Ahern D.J., Almuttaqi H., Alonzi D.S., Alrubayyi A., Alsaleh G., Bart V.M.T., Batchelor V. (2021). Mucosal immune responses in COVID19—A living review. Oxf. Open Immunol..

[4] Russell M.W., Moldoveanu Z., Ogra P.L., Mestecky J. (2020). Mucosal Immunity in COVID-19: A Neglected but Critical Aspect of SARS-CoV-2 Infection. Front. Immunol..

[5] Fröberg J., Diavatopoulos D.A. (2021). Mucosal immunity to severe acute respiratory syndrome coronavirus 2 infection. Curr. Opin. Infect. Dis..

[6] Li L., Wang M., Hao J., Han J., Fu T., Bai J., Tian M., Jin N., Zhu G., Li C. (2021). Mucosal IgA response elicited by intranasal immunization of Lactobacillus plantarum expressing surface-displayed RBD protein of SARS-CoV-2. Int. J. Biol. Macromol..

[7] Flament H., Rouland M., Beaudoin L., Toubal A., Bertrand L., Lebourgeois S., Rousseau C., Soulard P., Gouda Z., Cagninacci L. (2021). Outcome of SARS-CoV-2 infection is linked to MAIT cell activation and cytotoxicity. Nat. Immunol..

[8] Orellana B., Campaña O. (2020). Ear, nose and throat manifestations in patients with COVID-19. CIMEL.

[9] Bagga B., Cehelsky J.E., Vaishnaw A., Wilkinson T., Meyers R., Harrison L.M., Roddam P.L., Walsh E.E., DeVincenzo J.P. (2015). Effect of Preexisting Serum and Mucosal Antibody on Experimental Respiratory Syncytial Virus (RSV) Challenge and Infection of Adults. J. Infect. Dis..

[10] Deeks J.J., Dinnes J., Takwoingi Y., Davenport C., Spijker R., Taylor-Phillips S., Adriano A., Beese S., Dretzke J., di Ruffano L.F. (2020). Antibody tests for identification of current and past infection with SARS-CoV-2. Cochrane Infectious Diseases Group, ed. Cochrane Database Syst. Rev..

[11] Guo L., Ren L., Yang S., Xiao M., Chang D., Yang F., Cruz C.S.D., Wang Y., Wu C., Xiao Y. (2020). Profiling Early Humoral Response to Diagnose Novel Coronavirus Disease (COVID-19). Clin. Infect. Dis..

[12] Ma H., Zeng W., He H., Zhao D., Jiang D., Zhou P., Cheng L., Li Y., Ma X., Jin T. (2020). Serum IgA, IgM, and IgG responses in COVID-19. Cell. Mol. Immunol..

[13] Paces J., Strizova Z., Smrz D., Cerny J. (2020). COVID-19 and the Immune System. Physiol. Res..

[14] Khoshkam Z., Aftabi Y., Stenvinkel P., Lawrence B.P., Rezaei M.H., Ichihara G., Fereidouni S. (2021). Recovery scenario and immunity in COVID-19 disease: A new strategy to predict the potential of reinfection. J. Adv. Res..

[15] Brandtzaeg P. (2013). Secretory immunity with special reference to the oral cavity. J. Oral Microbiol..

[16] Varadhachary A. (2020). Salivary anti-SARS-CoV-2 IgA as an accessible biomarker of mucosal immunity against COVID-19. medRxiv.

[17] Aita A., Basso D., Cattelan A.M., Fioretto P., Navaglia F., Barbaro F., Stoppa A., Coccorullo E., Farella A., Socal A. (2020). SARS-CoV-2 identification and IgA antibodies in saliva: One sample two tests approach for diagnosis. Clin. Chim. Acta.

[18] Roda A., Cavalera S., Di Nardo F., Calabria D., Rosati S., Simoni P., Colitti B., Baggiani C., Roda M., Anfossi L. (2021). Dual lateral flow optical/chemiluminescence immunosensors for the rapid detection of salivary and serum IgA in patients with COVID-19 disease. Biosens. Bioelectron..

[19] To K.K.-W., Tsang O.T.-Y., Leung W.-S., Tam A.R., Wu T.-C., Lung D.C., Yip C.C.-Y., Cai J.-P., Chan J.M.-C., Chik T.S.-H. (2020). Temporal profiles of viral load in posterior oropharyngeal saliva samples and serum antibody responses during infection by SARS-CoV-2: An observational cohort study. Lancet Infect. Dis..

[20] Pisanic N., Randad P.R., Kruczynski K., Manabe Y.C., Thomas D.L., Pekosz A., Klein S.L., Betenbaugh M.J., Clarke W.A., Laeyendecker O. (2020). COVID-19 serology at population scale: SARS-CoV-2-specific antibody responses in saliva. J. Clin. Microbiol..

[21] Gordon C.J., Tchesnokov E.P., Woolner E., Perry J.K., Feng J.Y., Porter D.P., Götte M. (2020). Remdesivir is a direct-acting antiviral that inhibits RNA-dependent RNA polymerase from severe acute respiratory syndrome coronavirus 2 with high potency. J. Biol. Chem..

[22] Jiang S., Hillyer C., Du L. (2020). Neutralizing Antibodies against SARS-CoV-2 and Other Human Coronaviruses. Trends Immunol..

[23] Jiang S., Du L., Shi Z. (2020). An emerging coronavirus causing pneumonia outbreak in Wuhan, China: Calling for developing therapeutic and prophylactic strategies. Emerg. Microbes Infect..

[24] Samrat S.K., Tharappel A.M., Li Z., Li H. (2020). Prospect of SARS-CoV-2 spike protein: Potential role in vaccine and therapeutic development. Virus Res..

[25] Meng T., Cao H., Zhang H., Kang Z., Xu D., Gong H., Wang J., Li Z., Cui X., Xu H. (2020). The Insert Sequence in SARS-CoV-2 Enhances Spike Protein Cleavage by TMPRSS. bioRxiv.

[26] Du L., He Y., Zhou Y., Liu S., Zheng B.-J., Jiang S. (2009). The spike protein of SARS-CoV—A target for vaccine and therapeutic development. Nat. Rev. Microbiol..

[27] Galipeau Y., Greig M., Liu G., Driedger M., Langlois M.-A. (2020). Humoral Responses and Serological Assays in SARS-CoV-2 Infections. Front. Immunol..

[28] Infantino M., Pieri M., Nuccetelli M., Grossi V., Lari B., Tomassetti F., Calugi G., Pancani S., Benucci M., Casprini P. (2021). The WHO International Standard for COVID-19 serological tests: Towards harmonization of anti-spike assays. Int. Immunopharmacol..

[29] Infantino M., Manfredi M., Grossi V., Lari B., Fabbri S., Benucci M., Fortini A., Damiani A., Mobilia E.M., Panciroli M. (2021). Closing the serological gap in the diagnostic testing for COVID-19: The value of anti-SARS-CoV-2 IgA antibodies. J. Med. Virol..

[30] Anand P., Stahel V.P. (2021). The safety of Covid-19 mRNA vaccines: A review. Patient Saf. Surg..

[31] Lippi G., Henry B., Plebani M. (2021). Anti-SARS-CoV-2 Antibodies Testing in Recipients of COVID-19 Vaccination: Why, When, and How?. Diagnostics.

[32] Cristiano A., Nuccetelli M., Pieri M., Sarubbi S., Pelagalli M., Calugi G., Tomassetti F., Bernardini S. (2021). Serological anti-SARS-CoV-2 neutralizing antibodies association to live virus neutralizing test titers in COVID-19 paucisymptomatic/symptomatic patients and vaccinated subjects. Int. Immunopharmacol..

[33] Saad-Roy C.M., Morris S.E., Metcalf C.J.E., Mina M.J., Baker R.E., Farrar J., Holmes E.C., Pybus O.G., Graham A.L., Levin S.A. (2021). Epidemiological and evolutionary considerations of SARS-CoV-2 vaccine dosing regimes. Science.

[34] Park J.-G., Oladunni F.S., Rohaim M.A., Whittingham-Dowd J., Tollitt J., Hodges M.D., Fathallah N., Assas M.B., Alhazmi W., Almilaibary A. (2021). Immunogenicity and Protective Efficacy of an Intranasal Live-attenuated Vaccine Against SARS-CoV-2. iScience.

[35] Pereira N.L., Ahmad F., Byku M., Cummins N.W., Morris A.A., Owens A., Tuteja S., Cresci S. (2021). COVID-19: Understanding Inter-Individual Variability and Implications for Precision Medicine. Mayo Clin. Proc..

[36] Plotkin S.A. (2010). Correlates of Protection Induced by Vaccination. Clin. Vaccine Immunol..

[37] Isho B., Abe K.T., Zuo M., Jamal A.J., Rathod B., Wang J.H., Li Z., Chao G., Rojas O.L., Bang Y.M. (2020). Persistence of serum and saliva antibody responses to SARS-CoV-2 spike antigens in COVID-19 patients. Sci. Immunol..

[38] Ketas T.J., Chaturbhuj D., Portillo V.M.C., Francomano E., Golden E., Chandrasekhar S., Debnath G., Diaz-Tapia R., Yasmeen A., Kramer K.D. (2021). Antibody Responses to SARS-CoV-2 mRNA Vaccines Are Detectable in Saliva. Pathog. Immun..

[39] Mortari E.P., Russo C., Vinci M.R., Terreri S., Salinas A.F., Piccioni L., Alteri C., Colagrossi L., Coltella L., Ranno S. (2021). Highly Specific Memory B Cells Generation after the 2nd Dose of BNT162b2 Vaccine Compensate for the Decline of Serum Antibodies and Absence of Mucosal IgA. Cells.

[40] Fröberg J., Gillard J., Philipsen R., Lanke K., Rust J., van Tuijl D., Teelen K., Bousema T., Simonetti E., Jongh C. (2021). SARS-CoV-2 mucosal antibody development and persistence and their relation to viral load and COVID-19 symptoms. Nat. Commun..

[41] Wang Y., Zhang L., Sang L., Ye F., Ruan S., Zhong B., Song T., Alshukairi A.N., Chen R., Zhang Z. (2020). Kinetics of viral load and antibody response in relation to COVID-19 severity. J. Clin. Investig..

[42] Clements J.D., Freytag L.C. (2016). Parenteral Vaccination Can Be an Effective Means of Inducing Protective Mucosal Responses. Clin. Vaccine Immunol..

