# Correction: Guerrieri et al. Nasal and Salivary Mucosal Humoral Immune Response Elicited by mRNA BNT162b2 COVID-19 Vaccine Compared to SARS-CoV-2 Natural Infection. *Vaccines* 2021, *9*, 1499

**DOI:** 10.3390/vaccines11010172

**Published:** 2023-01-13

**Authors:** Mariapia Guerrieri, Beatrice Francavilla, Denise Fiorelli, Marzia Nuccetelli, Francesco Maria Passali, Luca Coppeta, Giuseppina Somma, Sergio Bernardini, Andrea Magrini, Stefano Di Girolamo

**Affiliations:** 1 Department of Otorhinolaryngology, University of Rome “Tor Vergata”, 00100 Rome, Italy; 2 Department of Experimental Medicine, University of Rome “Tor Vergata”, 00100 Rome, Italy; 3 Department of Occupational Medicine, University of Rome “Tor Vergata”, 00100 Rome, Italy; 4 Department of Laboratory Medicine, Tor Vergata University Hospital, 00100 Rome, Italy

## Error in Figure

The authors wish to make the following corrections to this paper [1]:

In the original publication, there was a mistake in Figure 2 as published. The same image was mistakenly selected for Figure 2 and Figure 3 during proofreading. The corrected Figure 2 appears below.

The authors apologize for any inconvenience caused and state that the scientific conclusions are unaffected. This correction was approved by the Academic Editor. The original publication has also been updated.

## Figures and Tables

**Figure 2:**
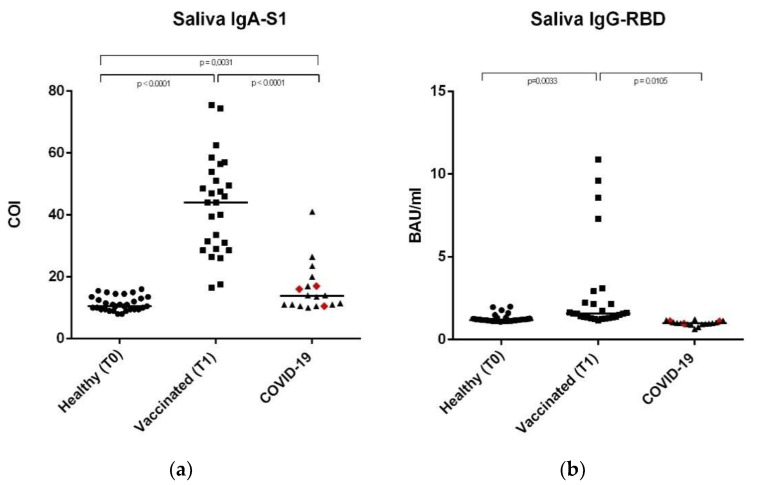
Anti-SARS-CoV-2 IgA-S1 and anti-SARS-CoV-2 IgG-RBD in saliva samples. (**a**) Saliva sample median levels of anti-SARS-CoV-2 IgA-S1 in the three study groups, expressed as COI (Cut off index). (**b**) Saliva sample median levels of anti-SARS-CoV-2 IgG-RBD in the three study groups, expressed as Binding Antibody Units (BAU/mL). In the COVID-19 group, the red rhombuses represent the hospitalized subjects. Statistical analysis and construction of figures were performed with GraphPad Prism 8 Software (GraphPad Software, San Diego, CA, USA). The D’Agostino and Pearson test, the Shapiro-Wilk normality test, and the Kolmogorov–Smirnov test were used to evaluate non-Gaussian distributions in all of the study populations. The continuous data were displayed as median and range. Non-parametric results were analysed with the Mann–Whitney test. For all results, *p* < 0.05 was considered statistically significant.
